# Pulsed-field ablation versus cryoballoon ablation in patients with persistent atrial fibrillation

**DOI:** 10.1016/j.ijcha.2025.101684

**Published:** 2025-04-30

**Authors:** Corinne Isenegger, Rebecca Arnet, Fabian Jordan, Sven Knecht, Philipp Krisai, Gian Völlmin, Jonas Brügger, David Spreen, Nicolas Schaerli, Behnam Subin, Beat Schär, Nicola Formenti, Felix Mahfoud, Christian Sticherling, Michael Kühne, Patrick Badertscher

**Affiliations:** a Department of Cardiology, University Hospital Basel, Basel, Switzerland; b Cardiovascular Research Institute Basel, University Hospital Basel, Basel, Switzerland

**Keywords:** Atrial fibrillation, Pulsed field ablation, Cryoballoon ablation, Pulmonary vein isolation

## Abstract

**Background:**

Current thermal energy sources such as cryoballoon (Cryo) ablation technology are associated with high rates of reconnected pulmonary veins (PV), especially in patients with persistent atrial fibrillation (AF). Pulsed-field ablation (PFA) may represent a more suitable ablation modality for this patient population. This study aims to compare the efficacy, and safety of PFA and Cryo in patients undergoing a PVI only approach for persistent AF.

**Method:**

Patients with persistent AF who underwent PVI at a tertiary referral center using either PFA or Cryo were consecutively enrolled.

**Results:**

A total of 220 patients (median age 66 [60–72] years, 24 % female) were included out of which 113 patients (51 %) underwent PFA and 107 patients (49 %) Cryoablation. Median procedure duration, LA dwell time and fluoroscopy time were shorter in the PFA group: 49 [39–61] min vs 60 [49–75] min (p < 0.001), 34 [25–43] min vs 37 [31––53] min (p < 0.001), and 9 [[8], [9], [10], [11], [12], [13]] min vs 11 [[8], [9], [10], [11], [12], [13], [14], [15], [16]] min (p = 0.008). During a median follow-up of 365 days, recurrence-free survival was 72 % in the PFA group and 60 % in the Cryo group (p_Log-rank_ = 0.079). The change in AF type from persistent AF to paroxysmal AF was more frequently observed after PFA than after Cryo (68 % vs 37 %; p = 0.011).

**Conclusion:**

In patients with persistent AF undergoing a PVI only approach, PFA was associated with shorter procedural times and similar efficacy, with a higher frequency of regression from persistent to paroxysmal AF. Future studies are needed to evaluate the role of ablation strategies beyond PVI when using PFA.

## Graphical abstract

**Central illustration:** Cryo = Cryoballoon ablation; LA = Left atrial; PFA = Pulsed-field ablation.

**Figure:**
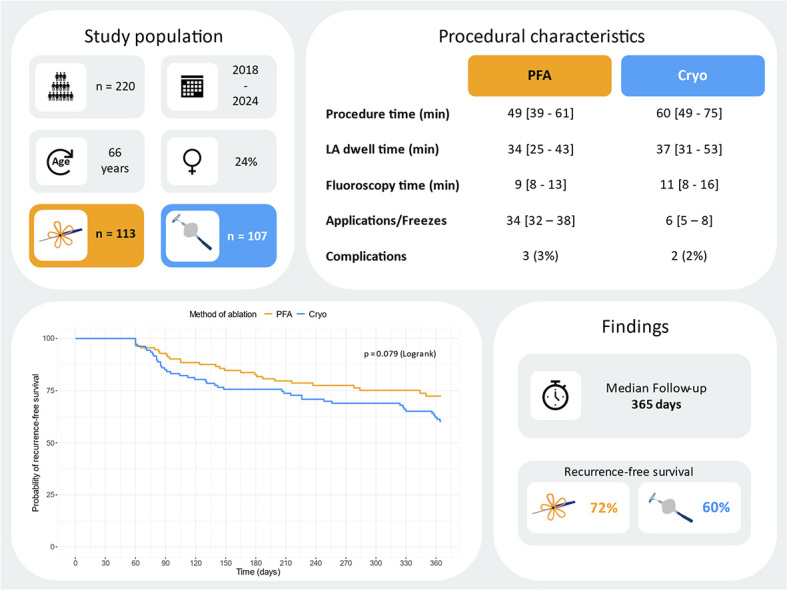

## 1 Introduction

Pulmonary vein isolation (PVI) is an effective treatment option for patients with atrial fibrillation (AF).[1,2] While the arrhythmia-free survival at 12 months after PVI in patients with paroxysmal AF is around 65–80 % [3], the procedural efficacy in patients with persistent AF is lower, with success rates around 55–60 % at 12 months follow-up [4,5].

The modest efficacy of PVI in persistent AF may be attributed to different mechanisms. Several ablation strategies beyond PVI, such as posterior wall isolation [6] or left atrial appendage isolation [7], have been investigated; however, none of these strategies showed a clear superiority in randomized studies. It remains unclear if differences in outcomes between patients with paroxysmal and persistent AF undergoing catheter ablation suggest that the mechanisms underlying the maintenance of persistent AF are different.

In both paroxysmal and persistent AF, the primary objective of catheter ablation is to achieve durable and homogenous isolation of the PV while minimizing the the risk of collateral thermal injury to adjacent structures. However, current thermal energy sources, including radiofrequency energy or cryoballoon (Cryo) ablation, are associated with rates of PV reconnection as high as 58 % in patients with persistent AF. Calvert et al. [8] recently showed a PV reconnection rate of 58 % in patients undergoing repeat invasive remapping after PVI for persistent AF, which may a main driver of arrhythmia recurrence in this challenging patient population.

Advancements in ablation technologies have introduced alternatives to traditional thermal methods, such as pulsed field ablation (PFA). A recent systematic review and *meta*-analysis assessing lesion durability after PVI demonstrated an 87 % durability rate for PFA lesions in mandatory remapping studies, suggesting improved outcomes compared to thermal techniques.[9].

Thus, PFA may represent the preferred modality for patients with persistent AF. While the safety, efficacy, and efficiency of PFA have been studied, primarily in patients with paroxysmal AF [10,11] or in combination with adjunctive lesions sets such posterior wall isolation[[12], [13], [14]] direct comparisons between PFA versus Cryo in patients with persistent AF undergoing a PVI-only approach are lacking.

This study aimed to compare PFA and Cryo in terms of procedural characteristics, safety, and efficacy in patients with persistent AF undergoing PVI only treatment.

## 2 Methods

### 2.1 Patient population

The study population includes patients with persistent AF, all of whom underwent their first PVI using either PFA or Cryo at the University Hospital Basel, Switzerland. These patients were consecutively enrolled in the prospective SWISS-AF-PVI registry (NCT03718364) between January 2018 and August 2024. Patients undergoing PVI only with no other additional lesion sets were analyzed. These patients were divided into two groups based on the energy source used: the PFA group and the Cryo group. This study was conducted in accordance with the Declaration of Helsinki.

### 2.2 Procedures

#### 2.2.1 Preprocedural management

All patients received transesophageal echocardiography the day before the procedure to rule out a left atrial thrombus. Preprocedural imaging of the left atrium, either through computed tomography or magnetic resonance imaging, was performed in each participant.

#### 2.2.2 Pulmonary vein isolation

The PVI was conducted by four experienced electrophysiologists. Procedures were performed under sedation using Midazolam, Fentanyl and Propofol. The femoral access was placed with ultrasound guidance. Fluoroscopic guidance or, in selected cases, intracardiac or transesophageal echocardiographic guidance was used for transseptal puncture. Heparin was administered with a target activated clotting time of 350 s. The intracardiac electrograms and surface electrograms were recorded at a speed of 100  mm/s (Sensis, Siemens, Erlangen, Germany).

#### 2.2.3 Pulsed field ablation

For PFA, the pentaspline ablation catheter (Farawave Boston Scientific, Natick, MA, USA) was used for PVI. In brief, after transseptal puncture, the PV was cannulated using a J-tip guidewire (Amplatz Extra-Stiff (0.035″)), and the pentaspline ablation catheter was positioned in the left atrium. The PVs were isolated using 4 PFA applications per PV with a voltage of 2 kV in both the basket configuration and the flower configuration. Additionally, patients received 2 PFA applications in flower configuration at the right-sided carina, as previously described [15]. The catheter was rotated by 30–40° after two consecutive applications in each configuration to ensure uniform coverage of the entire circumference.. The procedural endpoint was defined as PVI, assessed directly at the end of procedure for all PV using the pentaspline catheter. For endpoint confirmation, entrance and, if deemed necessary, e.g. due to residual far-field atrial signal, exit block was tested via the PFA catheter by pacing with with 5 V and a pulse-width of 1.0  ms ms among all electrodes or via a 3D-electroanatomical mapping system (EAM, CARTO3, Biosense Webster, Irvine, CA, USA).

#### 2.2.4 Cryoballon ablation

Cryo ablation was performed as previously described [16,17], using either the PolarX Cardiac Cryoablation System (Boston Scientific, Natick, MA, USA) or the Arctic Front Cryoablation System (Medtronic, Minneapolis, MN, USA). The Cryo ablation catheter was placed in the left atrium and the sheath and catheter were aligned to occlude the PV.. The freezing cycle length was 180 – 240 s. Freezing cycles were terminated early if phrenic nerve injury occurred or if the temperatures of − 60 °C (Arctic Front) or − 70 °C (PolarX) were reached. No additional “bonus” freezes were performed with any of the ablation systems once a PV was successfully isolated. An effective freeze was defined as disappearance of all local PV signals before 60 s or reaching a temperature of − 40 °C (Arctic Front) or − 50 °C (PolarX) within 60 s. Phrenic nerve injury (PNI) was monitored by continuously pacing the right phrenic nerve (PN) via the right subclavian vein during the freezing of the right pulmonary veins, and if a decrease in PN function (CMAP reduction > 30 %) was detected, the freezing process was immediately halted.[18] Phrenic nerve palsies were defined as instances where PNI remained at the conclusion of the procedure. The procedural endpoint was defined as PVI, assessed directly at the end of procedure for all PV. For endpoint confirmation, entrance and, if deemed necessary, e.g. due to residual far-field atrial signal, exit block was tested via the spiral mapping catheter by pacing with 10  V and a pulse-width of 1.0  ms among all electrodes. No 3D-EAM was used in the Cryo group.

#### 2.2.5 Post-ablation management

Oral anticoagulation was maintained for a minimum of 2 months following ablation. Antiarrhythmic therapy was continued for 3 months following ablation. Hemostasis was achieved using a figure-of-eight suture, followed by 4 h of bed rest. Transthoracic echocardiography was performed within one hour after the procedure to rule out pericardial effusion.

### 2.3 Biomarkers of myocardial injury

Fasting blood samples were obtained on the morning before the procedure and again the day after the procedure. High-sensitivity troponin T (hs-cTnT) levels were measured using the Roche Elecsys 2010 assay (Roche Diagnostics), which has a 99th percentile concentration of 14 ng/L and a coefficient of variation of 10 % at 13 ng/L[19].

### 2.4 Endpoints and follow-Up

The efficacy endpoint of this study was arrhythmia-free survival during the follow-up period. After a 60-day blanking period [20], any atrial arrhythmia, including AF, typical atrial flutter, atypical atrial flutter, or atrial tachycardia (AT) lasting at least 30 s and recorded on an ECG, was considered as a recurrence. If an arrhythmia occurred during the blanking period and persisted beyond the 60 days mark, the recurrence date was adjusted to the end of the blanking period. Secondary efficacy endpoints included the total duration of the procedure (from femoral puncture to sheath removal), left atrial (LA) dwell time, and first pass isolation (FPI). Procedural safety endpoints included phrenic nerve injury, pericardial tamponade, transient ischemic attack/stroke, or issues related to vascular access. The follow-up protocol involved scheduled outpatient visits at 3, 6, and 12 months, which included a physical examination, a 12-lead ECG, and a 7-day Holter ECG. The follow-up was limited to 1 year. If symptomatic arrhythmia occurred outside these planned visits, an additional 12-lead ECG or Holter ECG was conducted to document the arrhythmia.

### 2.5 Statistical analysis

The median and interquartile range (IQR) were used to describe continuous variables, and comparisons were made using the Wilcoxon rank sum test. Categorical variables were presented as numbers and percentages, and were compared using either the χ2 or Fisher’s exact test, as appropriate. Recurrence rates and FPI were compared using the Test of proportions. The Kaplan-Meier method, combined with a log-rank test, was utilized to assess and compare the probability of remaining free from atrial arrhythmia recurrence. A p-value of less than 0.05 was considered statistically significant. The analysis was conducted using R software (R Core Team, 2021, R Foundation for Statistical Computing, Vienna, Austria) and RStudio (RStudio Team, 2019, RStudio, Inc., Boston, MA, USA).

## 3 Results

### 3.1 Baseline characteristics

A total of 220 patients with persistent AF were included. Of these, the median age was 66 [60 – 72] years and 24 % were female. 113 patients (51 %) were in the PFA group and 107 (49 %) in the Cryo group. The baseline characteristics of the two groups are further summarized in Table 1.

**Table 1:** Patient characteristics of patients undergoing a PFA or Cryo ablation for PVI. PFA = Pulsed-field Ablation; Cryo = Cryoballoon ablation; AF = Atrial fibrillation; LA = Left atrial, LAVI = Left atrial volume index; LVEF = Left ventricular ejection fraction.

	**Overall** N = 220	**PFA** N = 113	**Cryo** N = 107	**p-value**
**Age, year**	66 [60–––72]	65 [59–––73]	67 [60–––71]	0.701
**Sex (female)**	53 (24 %)	25 (22 %)	28 (26 %)	0.483
**BMI, kg/m^2^**	28 [25], [26], [27], [28], [29], [30], [31]	27 [25], [26], [27], [28], [29], [30], [31]	28 [25], [26], [27], [28], [29], [30], [31]	0.724
**LVEF, %**	55 [47–––60]	54 [45–––59]	56 [50–––61]	0.048
**LA diameter, mm**	43 [39–––46]	43 [39–––46]	42 [39–––47]	0.846
**CHADS2VASC-Score**	2 [1], [2], [3]	2 [1], [2], [3]	3 [1], [2], [3]	0.153
**EHRA score**				0.874
I	48 (27 %)	32 (29 %)	16 (23 %)	
IIa	48 (27 %)	28 (25 %)	20 (29 %)	
IIb	55 (31 %)	33 (30 %)	22 (31 %)	
III	28 (16 %)	16 (15 %)	12 (17 %)	
IV	1 (1 %)	1 (1 %)	0 (0 %)	
**Hypertension**	137 (62 %)	67 (59 %)	70 (65 %)	0.349
**Diabetes**	20 (9 %)	11 (10 %)	9 (8 %)	0.733
**Hypercholesterolemia**	65 (35 %)	39 (35 %)	26 (35 %)	0.983
**Coronary artery disease**	24 (11 %)	12 (11 %)	12 (11 %)	0.887
**Smoking history**	115 (56 %)	61 (56 %)	54 (56 %)	0.966

### 3.2 Procedural characteristics

The median procedure duration was 49 [39 – 61] min vs. 60 [49 – 75] min, p < 0.001, the LA dwell time was 34 [25 – 43] min vs. 37 [31 – 53] min, p < 0.001, and the fluoroscopy time was 9 [8], [9], [10], [11], [12], [13] min vs. 11 [8], [9], [10], [11], [12], [13], [14], [15], [16] min, p = 0.008, for the PFA group vs. Cryo group, respectively. Periprocedural EAM was performed in 79 patients (70 %) of the PFA group and in 1 patients (1 %) of the Cryo group (p < 0.001). When comparing only patients who received PVI without EAM (Table S1), the results for shorter procedural durations in the entire study population were confirmed, except that fluoroscopy time was similar between the PFA and the Cryo group: 10 [9], [10], [11], [12], [13] min vs. 11 [8], [9], [10], [11], [12], [13], [14], [15], [16] min, p = 0.248. Hs-cTnT one day after PVI reflecting the myocardial injury, was significantly higher in the PFA group compared to the Cryo group: 1078 [822 – 1519] ng/l vs 776 [579 – 1065] ng/l, p < 0.001. Procedural characteristics are summarized in Table 2.

**Table 2:** Comparing procedural characteristics of PVI using PFA or Cryo. The ablation time is defined as the time from first ablation to last ablation. PFA = Pulsed-field ablation; PVI = Pulmonary vein isolation; LA = Left atrial; AF = Atrial fibrillation; SR = Sinus Rhythm; Cryo = Cryoballoon Ablation; Hs-cTnT = high-sensitive cardiac troponin T.

**Procedural characteristics**	**PFA** N = 113	**Cryo** N = 107	**p-value**
**Total procedure duration, min**	49 [39–––61]	60 [49–––75]	**<0.001**
**LA Dwell time, min**	34 [25–––43]	37 [31–––53]	**<0.001**
**Ablation time, min**	17 [11], [12], [13], [14], [15], [16], [17], [18], [19], [20], [21]	31 [26–––42]	**<0.001**
**Fluoroscopy time, min**	9 [8], [9], [10], [11], [12], [13]	11 [8], [9], [10], [11], [12], [13], [14], [15], [16]	**0.008**
**Fluoroscopy dose, Gycm^2^**	454 [225–––857]	410 [225–––809]	0.833
**Rhythm before ablation**			**0.029**
AF	76 (67 %)	44 (52 %)	
SR	37 (33 %)	41 (48 %)	
**Applications/Freezes**	34 [32–––38]	6 [5], [6], [7], [8]	**<0.001**
**Electroanatomic Mapping System**	79 (70 %)	0 (0 %)	**<0.001**
**Hs-cTnT prior to PVI, ng/L**	10 [8], [9], [10], [11], [12], [13], [14]	10 [7], [8], [9], [10], [11], [12], [13], [14]	0.977
**Hs-cTnT 1 day after PVI, ng/L**	1078 [822–––1519]	776 [579–––1065]	**<0.001**

### 3.3 Procedural endpoints and safety

Acute PVI was achieved in 100 % of cases. Overall, complications occurred in 5 cases (2 %) across the study population. Specifically, 3 complications in 2 patients (2 %) were observed in the PFA group, including 2 cases of stroke/TIA and 1 case of pericardial tamponade, treated with drainage. One case of stroke occurred 2 days after PVI and resulted in mild ataxia and reduced fine motor skills of the left hand, both of which persisted at discharge and during 6-months follow-up. The other case experienced transient diplopia for 6 h post-procedure, was admitted to the stroke unit, and discharged home without any residual symptoms. In the Cryo group, a total of 2 complications (2 %) were noted with 2 cases of phrenic nerve palsies. One case of phrenic nerve palsy persisted at discharge, while the other case was asymptomatic upon discharge. Both phrenic nerve palsies recovered fully during follow-up.

### 3.4 Arrhythmia-free survival and follow-up

**Fig. 1:**
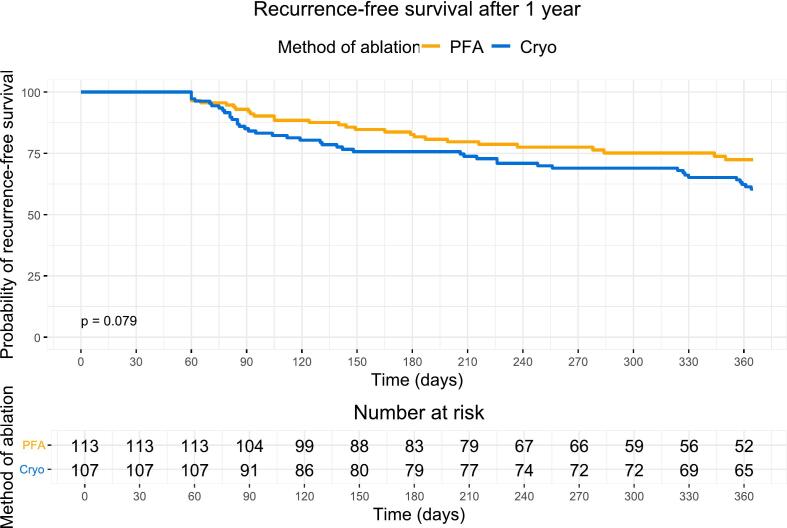
Kaplan-Meier curve comparing probability of recurrence-free survival over 1 year between the PFA group and the Cryo group. Cryo = Cryoballoon ablation; PFA = Pulsed-field ablation.

During a median follow-up of 365 days [155 – 365] days, the Kaplan-Meier analysis estimated a recurrence-free survival of 72 % (95 % CI 64 % – 82 %) in the PFA group and 60 % (95 % CI 52 % – 70 %) in the Cryo group (p_Log-rank_ = 0.079) (Fig. 1) (Table 3). This result was consistent when excluding patients on AAD treatment at any time during follow-up (Fig. S1). Among 70 patients (32 %) with recurrent atrial arrhythmia, 28 (25 %) occurred in the PFA group and 42 (39 %) in the Cryo group. The recurrence rates of AF, Aflu, and AT were similar in both groups: Of the patients with recurrent arrhythmia in the PFA group, 26 patients (93 %) had recurrent AF, 1 patient (4 %) had Aflu and 1 patient (4 %) had AT. Of the patients with recurrent arrhythmia in the Cryo group, recurrent AF was observed in 35 patients (83 %), Aflu in 1 patient (2 %) and AT in 6 patients (14 %). Reassessment and reclassification of the type of AF during follow-up revealed a more frequent change from persistent to paroxysmal AF after PFA than after Cryo (68 % vs 37 %; p = 0.011). Similar results were found when comparing the recurrence-free survival of patients undergoing PVI without the use of an EAM with no difference between the PFA and the Cryo group (62 % vs 60 %, p_Log-rank_ = 0.660) (Fig. S2).

**Table 3:** Safety and efficacy in patients undergoing their first PFA or Cryo catheter ablation. AF = Atrial fibrillation; AFlu = Atrial Flutter; AT = Atrial tachycardia; Cryo = Cryoballoon ablation; PFA = Pulsed-field ablation; PV = Pulmonary vein.

**Efficacy and Safety**	**Overall** N = 220	**PFA** N = 113	**Cryo** N = 107	**p-value**
**Recurrence**	68 (31 %)	28 (25 %)	42 (39 %)	**0.021**
**Recurrence of any atrial arrhythmia**				0.344
AF	59 (87 %)	24 (92 %)	35 (83 %)	
AFlu	2 (3 %)	1 (4 %)	1 (2 %)	
AT	7 (10 %)	1 (4 %)	6 (14 %)	
**Paroxysmal AF after recurrence**	34 (49 %)	19 (68 %)	15 (37 %)	**0.011**
**Patients undergoing redo procedure**	41 (60 %)	10 (38 %)	31 (74 %)	**0.004**
**Redo procedure within first year**	23 (34 %)	6 (21 %)	17 (40 %)	0.096
**Number of reconnected PVs**				**0.023**
0	9 (22 %)	5 (50 %)	4 (13 %)	
1	9 (22 %)	0 (0 %)	9 (29 %)	
≥2	23 (56 %)	5 (50 %)	18 (58 %)	
**Reconnected RSPV**	18 (45 %)	4 (40 %)	14 (47 %)	>0.999
**Reconnected RIPV**	14 (35 %)	4 (40 %)	10 (33 %)	0.718
**Reconnected LSPV**	15 (38 %)	2 (20 %)	13 (43 %)	0.269
**Reconnected LIPV**	13 (33 %)	2 (20 %)	11 (37 %)	0.451
**Complications**	5 (2 %)	3 (3 %)	2 (2 %)	>0.999
**Type of complications**				0.179
Pericardial tamponade	1 (0.5 %)	1 (1 %)	0 (0 %)	
Phrenic nerve injury	2 (1 %)	0 (0 %)	2 (2 %)	
Stroke	2 (1 %)	2 (2 %)	0 (0 %)	

### 3.5 Repeat procedures

Among the 70 patients (32 %) with recurrent atrial arrhythmias, 41 patients (60 %) underwent repeat catheter ablation, 10 (24 %) in the PFA group and 31 (76 %) in the Cryo group. Repeat procedures within the first year were performed in 21 % in the PFA group and in 40 % in the Cryo group, p = 0.096. Durably isolated PVs were noted in 50 % of patients in the PFA group and in 13 % of patients in the Cryo group, p = 0.023. Of a total of 39 remapped PVs in the PFA group, 12 PVs (31 %) were reconnected. In the Cryo group, 48 out of 123 PVs (39 %) were reconnected. In the PFA group, the most frequently reconnected PVs were the right superior PV (RSPV), and the right inferior PV (RIPV), each with 40 %, followed by the left superior PV (LSPV), and the left inferior PV (LIPV), each with 20 %. In the Cryo group, the most frequent reconnected PV was the RSPV in 47 %, followed by LSPV in 43 %, the LIPV in 37 % and the RIPV in 33 %.

## 4 Discussion

This study aimed to assess and compare the efficacy, safety, and efficacy of PFA and Cryo in patients undergoing a PVI only approach for persistent AF. Our main findings were as follows:

First, procedural times were significantly shorter for the PFA than for the Cryo group with a procedure duration of 49 min, a LA dwell time of 34 min, and a fluoroscopy time of 9 min. These results are consistent with previous studies reporting reduced procedural times for PFA compared to Cryo in patients with paroxysmal AF.[21], [22] Second, both PFA and Cryo demonstrated excellent safety profiles.[21], [22], [23] Third, in patients with recurrent atrial arrhythmias undergoing repeat catheter ablation, PFA demonstrated significantly more durably isolated PVs. Reconnected PVs were commonly observed on the right side, with 67 % in the PFA and 50 % in the Cryo groups, respectively. This discrepancy may be explained by the anatomical proximity of the transseptal puncture to the RIPV, which requires acute angles of the ablation catheter and the generally larger diameter of the RSPV compared to the other PVs.[24] These findings highlights the need for further optimization of catheter designs for both single-shot modalities. Fourth, at 365 days of follow-up, recurrence-free survival rate was 72 % for the PFA group and 60 % for the Cryo group. Additionally, the recurrence-free survival rate was higher in the PFA group (72 %) compared to the Cryo group (60 %). Additionally, patients undergoing PFA experienced a higher rate of transition from persistent AF to paroxysmal AF, with a conversion rate of 68 % versus 37 % for Cryo (p = 0.011).

These findings corroborate and extend with previous studies investing PVI in patients with persistent AF in terms recurrence-free survival rates for PFA [21], [25], [26], [27] as well as for Cryo [23], [28], [29]. Mililis et al. [30] performed a 2:1 randomized trial in 199 patients with persistent AF undergoing PVI only using radiofrequency ablation (RFA) vs. Cryo ablation and reported similar recurrence-free survival rates between RFA and Cryo (65 % vs 62 %) during 12 months of follow-up. Although evidence for PFA in persistent AF is scarce, a study by Kueffer et al.[22] compared PFA, Cryo and RFA in patients with persistent AF. They included 214 patients in the PFA group, 190 in the Cryo group and 129 in the RFA group. While a PVI-only approach was used in the Cryo and RFA group, one fifth of patients in the PFA group received additional posterior wall ablation (PWI). After 12-month follow-up, freedom from AF recurrence was 55 % in the PFA group, 62 % in the Cryo group and 48 % in RF group, with improved arrhythmia-free survival observed in both the PFA and Cryo groups compared to RFA (PFA vs RFA: *P* = 0.010; CBA vs RFA: p = 0.009). Interestingly, when excluding patients with additional PWI in the PFA group, 1-year freedom was only 48 %.

However, as PFA is a relatively new procedure, there is no data yet on long-term outcomes. In comparison, Heeger et al. [31] published a 5-year outcome where patients with persistent AF (n = 34) had a recurrence-free survival rate of 46.9 %.

This study highlights the potential advantages of PFA in procedural efficacy and PV durability. However, it also emphasizes the need for continued exploration of additional ablation strategies in patients with persistent AF, e.g. additional trigger and substrate ablations such as PWI. PFA could be more effective than RFA for PWI because of more extensive and durable ablation achieved by PFA. Ongoing research, including the PIFPAF-PFA trial (NCT05986526) which will randomize patients with persistent AF to PVI alone or PVI combined with PWI, is expected to provide critical insights into the optimal use of PFA in this patient population.

### 4.1 Limitations

This study has several limitations. First, this is a non-randomized, observational study, which inherently limits the ability to draw definitive conclusions.. Second, not all patients received continuous monitoring with an implantable loop recorder during the follow-up period, potentially leading to underestimation of arrhythmia recurrence. Third, intracardiac echocardiography (ICE) for direct visualization of the PFA catheter was not routinely performed. Although recent evidence suggests no significant advantage to ICE [32], it remains unclear whether outcomes would have been different when using ICE. Fourth, there was no standard protocol regarding the indication for repeat procedures.

## 5 Conclusion

This study demonstrated that in patients with persistent AF undergoing a PVI only approach, PFA is associated with shorter procedural times, similar efficacy, and a higher rate transition from persistent to paroxysmal AF compared to Cryo. Furthermore, PFA resulted in significantly more durably isolated PVs in patients undergoing repeat catheter ablation. Future studies in this challenging patient population assessing the role of ablation of targets beyond PVI when using PFA are needed.

**Statement of authorship:** All authors take responsibility for all aspects of the reliability and freedom from bias of the data presented and their discussed interpretation.

## Disclosures

Patrick Badertscher has received research funding from the “University of Basel“, the “Stiftung für Herzschrittmacher und Elektrophysiologie”, the “Freiwillige Akademische Gesellschaft Basel”, the “Swiss Heart Foundation” and Johnson&Johnson, and reports personal fees from BMS, Boston Scientific and Abbott, all outside the submitted work.

Philipp Krisai reports speaker fees from BMS/10.13039/100004319Pfizer. Grants from the 10.13039/501100001711Swiss National Science Foundation, 10.13039/501100004362Swiss Heart Foundation, 10.13039/100007555Foundation for Cardiovascular Research Basel, Machaon Foundation.

Sven Knecht has received funding the “Swiss Heart Foundation”.

Felix Mahfoud has been supported by Deutsche Gesellschaft für Kardiologie (DGK), Deutsche Forschungsgemeinschaft (SFB TRR219, Project-ID 322900939), and Deutsche Herzstiftung. Until May 2024, FM has received speaker honoraria/consulting fees from Ablative Solutions, Amgen, Astra-Zeneca, Bayer, Boehringer Ingelheim, Inari, Medtronic, Merck, ReCor Medical, Servier, and Terumo all outside the submitted work.

Christian Sticherling: Member of Medtronic Advisory Board Europe and Boston Scientitic Advisory Board Europe, received educational grants from Biosense Webster and Biotronik and a research grant from the European Union’s FP7 program and Biosense Webster and lecture and consulting fees from Abbott, Medtronic, Biosense-Webster, Boston Scientific, Microport, and Biotronik all outside the submitted work.

Michael Kühne reports grants from the Swiss National Science Foundation (Grant numbers 33CS30_148474, 33CS30_177520, 32473B_176178, 32003B_197524), the Swiss Heart Foundation, the Foundation for Cardiovascular Research Basel and the University of Basel, grants from Bayer, grants from Pfizer, grants from Boston Scientific, grants from BMS, grants from Biotronik, grants and personal fees from Daiichi Sankyo, all outside the submitted work.

Behnam Subin reports a travel grant from Boston Scientific.

## CRediT authorship contribution statement

**Corinne Isenegger:** Writing – original draft, Visualization, Formal analysis, Data curation. **Rebecca Arnet:** Writing – review & editing. **Fabian Jordan:** Writing – review & editing, Validation. **Sven Knecht:** Writing – review & editing, Data curation. **Philipp Krisai:** Writing – review & editing, Investigation. **Gian Völlmin:** Project administration, Investigation, Data curation. **Jonas Brügger:** Writing – review & editing, Validation. **David Spreen:** Writing – review & editing, Investigation. **Nicolas Schaerli:** Writing – review & editing, Investigation. **Behnam Subin:** Writing – review & editing, Investigation. **Beat Schär:** Writing – review & editing. **Nicola Formenti:** Writing – review & editing, Data curation. **Felix Mahfoud:** Writing – review & editing. **Christian Sticherling:** Writing – review & editing, Investigation. **Michael Kühne:** Writing – review & editing, Supervision, Project administration, Methodology, Investigation, Conceptualization. **Patrick Badertscher:** Writing – original draft, Supervision, Methodology, Investigation, Conceptualization.

## Funding

Swiss Heart Foundation, University of Basel.

## Declaration of competing interest

The authors declare the following financial interests/personal relationships which may be considered as potential competing interests: [Patrick Badertscher reports a relationship with University of Basel that includes: funding grants. Patrick Badertscher reports a relationship with Stiftung für Herzschrittmacher und Elektrophysiologie that includes: funding grants. Patrick Badertscher reports a relationship with Freiwillige Akademische Gesellschaft Basel that includes: funding grants. Patrick Badertscher reports a relationship with Schweizerische Herzstiftung that includes: funding grants. Patrick Badertscher reports a relationship with Johnson & Johnson that includes: funding grants. Patrick Badertscher reports a relationship with Bristol Myers Squibb Co that includes: speaking and lecture fees. Patrick Badertscher reports a relationship with Boston Scientific Corporation that includes: speaking and lecture fees. Patrick Badertscher reports a relationship with Abbott that includes: speaking and lecture fees. Philipp Krisai reports a relationship with BMS-Pfizer that includes:. Philipp Krisai reports a relationship with Swiss National Science Foundation that includes: funding grants. Philipp Krisai reports a relationship with Schweizerische Herzstiftung that includes: funding grants. Philipp Krisai reports a relationship with Foundation for Cardiovascular Research Basel that includes: funding grants. Philipp Krisai reports a relationship with Machaon Foundation that includes: funding grants. Sven Knecht reports a relationship with Schweizerische Herzstiftung that includes: funding grants. Felix Mahfoud reports a relationship with German Cardiac Society that includes: funding grants. Felix Mahfoud reports a relationship with Deutsche Forschungsgemeinschaft that includes: funding grants. Felix Mahfoud reports a relationship with German Heart Foundation that includes: funding grants. Felix Mahfoud reports a relationship with Ablative Solutions Inc that includes: consulting or advisory and speaking and lecture fees. Felix Mahfoud reports a relationship with Amgen that includes: consulting or advisory and speaking and lecture fees. Felix Mahfoud reports a relationship with Astra-Zeneca that includes: consulting or advisory and speaking and lecture fees. Felix Mahfoud reports a relationship with Bayer that includes: consulting or advisory and speaking and lecture fees. Felix Mahfoud reports a relationship with Boehringer Ingelheim that includes: consulting or advisory and speaking and lecture fees. Felix Mahfoud reports a relationship with Inari that includes: consulting or advisory and speaking and lecture fees. Felix Mahfoud reports a relationship with Medtronic that includes: consulting or advisory and speaking and lecture fees. Felix Mahfoud reports a relationship with Merck that includes: consulting or advisory and speaking and lecture fees. Felix Mahfoud reports a relationship with ReCor Medical that includes: consulting or advisory and speaking and lecture fees. Felix Mahfoud reports a relationship with Servier that includes: consulting or advisory and speaking and lecture fees. Felix Mahfoud reports a relationship with Terumo that includes: consulting or advisory and speaking and lecture fees. Christian Sticherling reports a relationship with Medtronic Advisory Board Europe that includes: board membership. Christian Sticherling reports a relationship with Boston Scientitic Advisory Board Europe that includes: board membership. Christian Sticherling reports a relationship with Biosense Webster that includes: consulting or advisory, funding grants, and speaking and lecture fees. Christian Sticherling reports a relationship with Biotronik that includes: consulting or advisory, funding grants, and speaking and lecture fees. Christian Sticherling reports a relationship with European Union’s FP7 program that includes: funding grants. Christian Sticherling reports a relationship with Abbott that includes: consulting or advisory and speaking and lecture fees. Christian Sticherling reports a relationship with Medtronic that includes: consulting or advisory and speaking and lecture fees. Christian Sticherling reports a relationship with Boston Scientific that includes: consulting or advisory and speaking and lecture fees. Christian Sticherling reports a relationship with Microport that includes: consulting or advisory and speaking and lecture fees. Michael Kuehne reports a relationship with Swiss National Science Foundation that includes: funding grants. Michael Kuehne reports a relationship with Schweizerische Herzstiftung that includes: funding grants. Michael Kuehne reports a relationship with Foundation for Cardiovascular Research Basel that includes: funding grants. Michael Kuehne reports a relationship with Pfizer that includes: funding grants. Michael Kuehne reports a relationship with Boston Scientific that includes: funding grants. Michael Kuehne reports a relationship with Bristol Myers Squibb Co that includes: funding grants. Michael Kuehne reports a relationship with Biotronik that includes: funding grants. Michael Kuehne reports a relationship with Daiichi Sankyo that includes: funding grants and speaking and lecture fees. Behnam Subin reports a relationship with Boston Scientific that includes: travel reimbursement. If there are other authors, they declare that they have no known competing financial interests or personal relationships that could have appeared to influence the work reported in this paper].
