# Supplementary material for: Pulsed-field ablation versus cryoballoon ablation in patients with persistent atrial fibrillation
Source: Int J Cardiol Heart Vasc. 2025 Apr 30;59:101684. doi: 10.1016/j.ijcha.2025.101684 (PMC12076779; doi:10.1016/j.ijcha.2025.101684)
Supplement: Supplementary Data 1 [file mmc1.docx]

Supplementary Appendix

Table of contents

[Figure S1 2](#_Toc171504518)

[Figure S2 3](#_Toc171504519)

[Table S1 4](#_Toc171504520)

# Figure S1
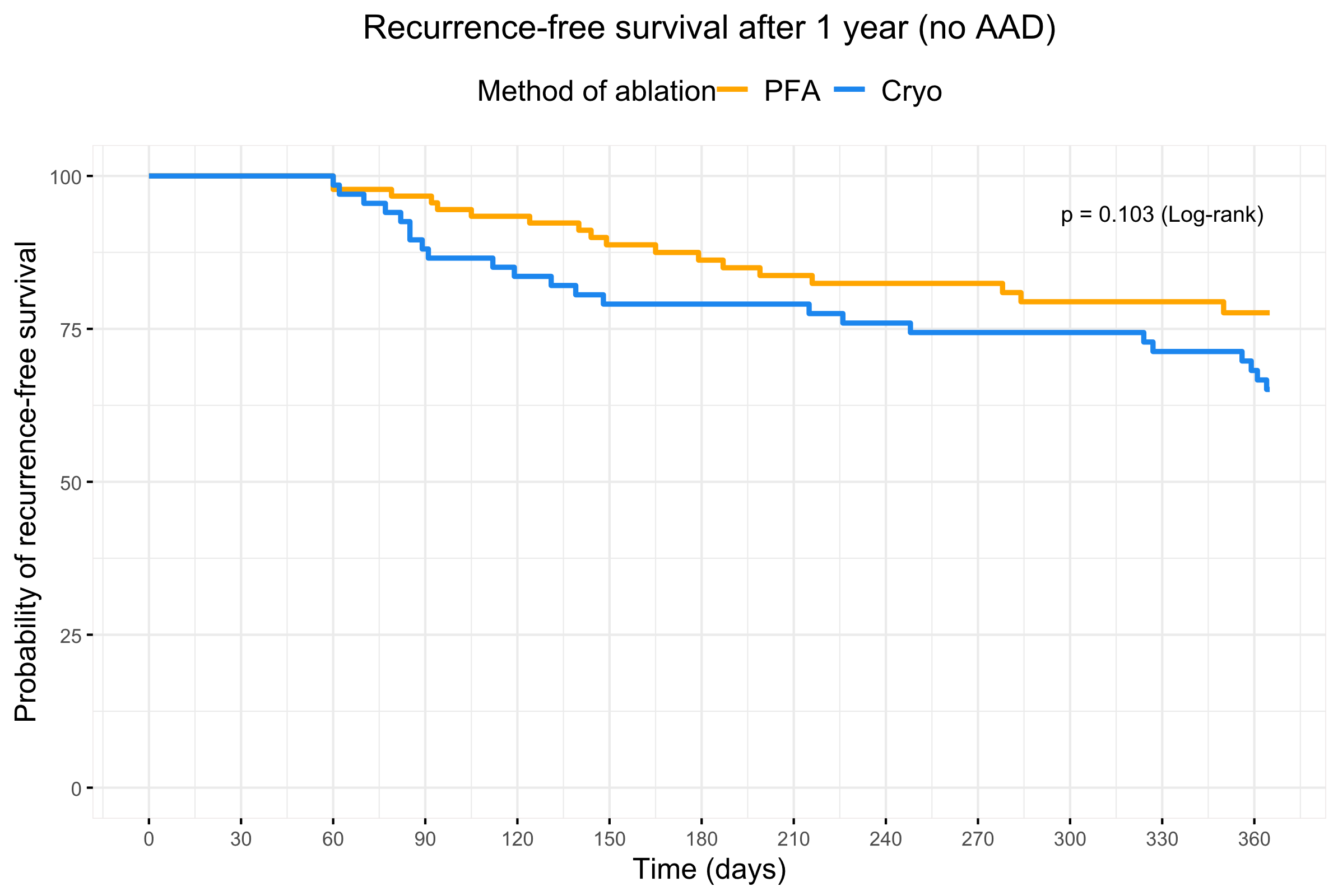


**Figure S1**: Subgroup analysis excluding all patients who received an AAD at any time: Kaplan-Meier curve comparing the probability of recurrence-free survival over 1 year between the PFA and Cryo groups. The recurrence-free survival rate was 77% for the PFA group (n=91) and 65% for the Cryo group (n=67) at a follow-up of 365 days. AAD = Anti arrhythmic drug; Cryo = Cryoballoon ablation; PFA = Pulse-field ablation.

# Figure S2
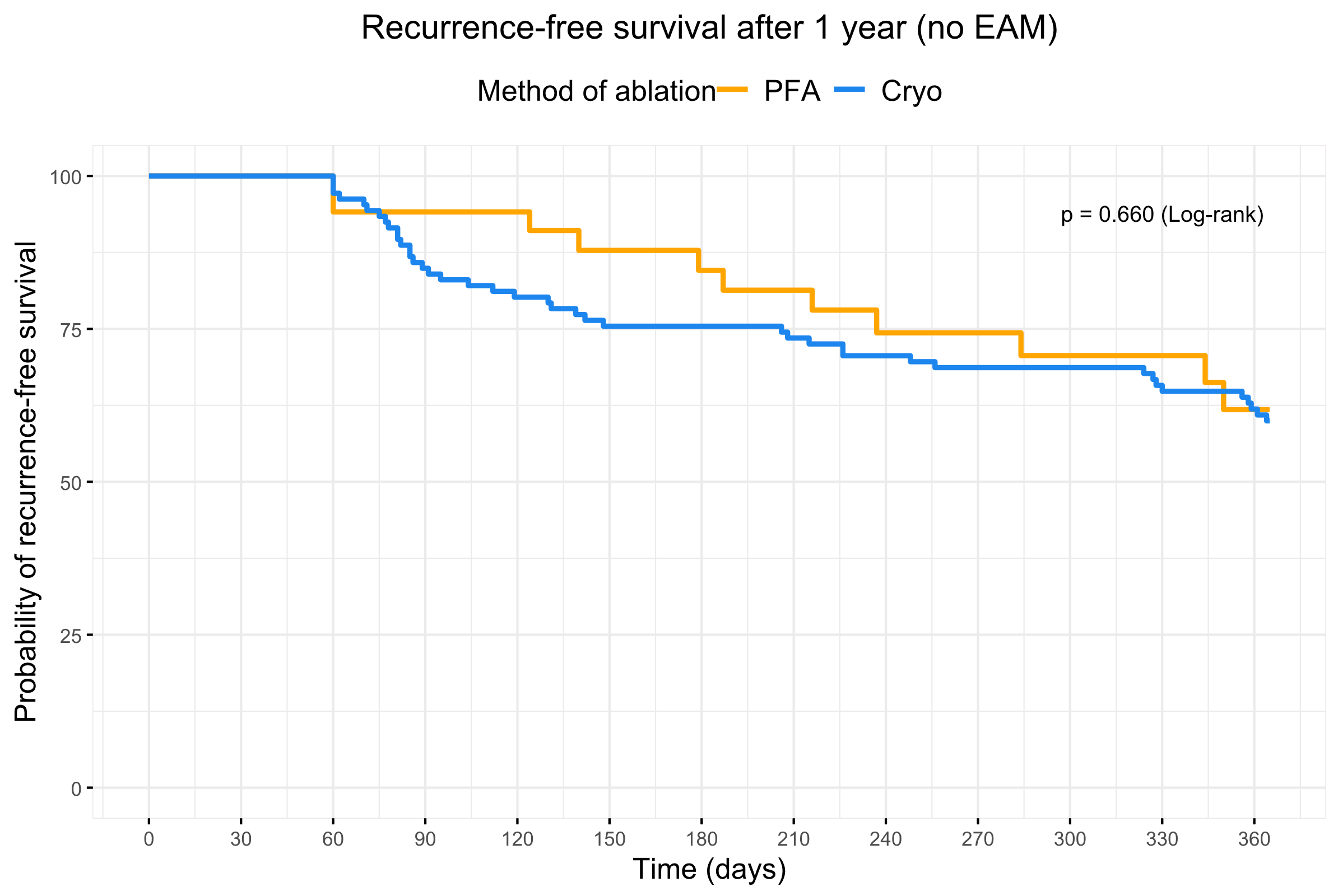


**Figure S2**: Subgroup analysis of patients undergoing PVI without the use of an 3D-EAM: Kaplan-Meier curve comparing the probability of recurrence-free survival over 1 year between the PFA and Cryo groups. The recurrence-free survival rate was 62% for the PFA group (n=34) and 61% for the Cryo group (n=106) at a follow-up of 365 days. Cryo = Cryoballoon ablation; 3D-EAM = 3D electroanatomical mapping; PFA = Pulse-field ablation; PVI = Pulmonary vein isolation.

# Table S1

| **Procedural characteristics** | **PFA** N = 34 | **Cryo** N = 106 | **p-value** |
| --- | --- | --- | --- |
| **Total procedure duration** | 36 [31 - 51] | 60 [49 - 75] | **<0.001** |
| **LA dwell time, min** | 23 [18 - 32] | 37 [31 - 49] | **<0.001** |
| **Ablation time, min** | 17 [12 - 20] | 31 [26 - 42] | **<0.001** |
| **Fluoroscopy time, min** | 10 [9 - 13] | 11 [8 - 16] | 0.248 |
| **Fluoroscopy dose, Gycm2** | 352 [147 - 690] | 406 [225 - 779] | 0.217 |
| **Rhythm before ablation** |  |  | 0.839 |
| AF | 19 (56%) | 44 (52%) |  |
| SR | 15 (44%) | 41 (48%) |  |
| **Hs-cTnT prior to PVI, ng/L** | 11 [7 - 15] | 10 [7 - 14] | 0.604 |
| **Hs-cTnT 1 day after PVI, ng/L** | 1085 [853 - 1718] | 776 [579 - 1065] | **<0.001** |

**Table S1**: Patient characteristics of the subgroup analysis of patients who underwent PVI without 3D EAM. AF = Atrial fibrillation; Cryo = Cryoballoon ablation; LA = Left atrial; Hs-cTnT = high-sensitive cardiac troponin T; PFA = Pulsed-field ablation; RFA = Radiofrequency ablation; SR = Sinus rhythm.
